# Heterologous Boosting With *Listeria*-Based Recombinant Strains in BCG-Primed Mice Improved Protection Against Pulmonary Mycobacterial Infection

**DOI:** 10.3389/fimmu.2020.02036

**Published:** 2020-09-02

**Authors:** Si-Jing Liu, Si-Cheng Tian, Yun-Wen Zhang, Tian Tang, Ju-Mei Zeng, Xiao-Yong Fan, Chuan Wang

**Affiliations:** ^1^West China School of Public Health and West China Fourth Hospital, Sichuan University, Chengdu, China; ^2^Food Safety Monitoring and Risk Assessment Key Laboratory of Sichuan Province, Department of Public Health Laboratory Sciences, West China School of Public Health, Sichuan University, Chengdu, China; ^3^Shanghai Public Health Clinical Center, Key Laboratory of Medical Molecular Virology of MOE/MOH, Fudan University, Shanghai, China

**Keywords:** *Mycobacterium tuberculosis*, vaccine, *Listeria monocytogenes*, *Listeria ivanovii*, multistage

## Abstract

While Baccillus Calmette-Guerin (BCG) is used worldwide, tuberculosis (TB) is still a global concern due to the poor efficacy of BCG. Novel vaccine candidates are therefore urgently required. In this study, two attenuated recombinant *Listeria* strains, LMΔ*-msv* and LIΔ*-msv* were constructed by deletion of *actA* and *plcB* and expression of a fusion protein consisting of T cell epitopes from four *Mycobacterium tuberculosis* (*Mtb*) antigens (*Rv2460c, Rv2660c, Rv3875*, and *Rv3804c*). The safety and immunogenicity of the two recombinant strains were evaluated in C57BL/6J mice. After intravenous immunization individually, both recombinant strains entered liver and spleen but eventually were eliminated from these organs after several days. Simultaneously, they induced antigen-specific cell-mediated immunity, indicating that the recombinant *Listeria* strains were immunogenic and safe *in vivo*. LMΔ*-msv* immunization induced stronger cellular immune responses than LIΔ*-msv* immunization, and when boosted with LIΔ*-msv*, antigen-specific IFN-γ CD8^+^ T cell responses were notably magnified. Furthermore, we evaluated the protection conferred by the vaccine candidates against mycobacterial infection via challenging the mice with 1 × 10^7^ CFU of BCG. Especially, we tested the feasibility of application of them as heterologous BCG supplement vaccine by immunization of mice with BCG firstly, and boosted with LMΔ*-msv* and LIΔ*-msv* sequentially before challenging. Combination immune strategy (LMΔ*-msv* prime-LIΔ*-msv* boost) conferred comparable protection efficacy as BCG alone. More importantly, BCG-vaccinated mice acquired stronger resistance to Mycobacterial challenge when boosted with LMΔ*-msv* and LIΔ*-msv* sequentially. Our results inferred that heterologous immunization with *Listeria*-based recombinant strains boosted BCG-primed protection against pulmonary mycobacterial infection.

## Introduction

Tuberculosis (TB) is one of the important global health problems caused by *Mycobacterium tuberculosis* (*Mtb*) complex. Bacille Calmette-Guerin (BCG) is the only licensed vaccine for TB, which efficiently protects against severe childhood forms of tuberculosis, but its protection efficacy persists for only about 15 years (1). Its protective efficacy against pulmonary TB in adults is variable ranging from 0 to 77%, and might be insufficient to control the reactivation of latent tuberculosis infection (LTBI) (1, 2). Considering the protective efficacy of BCG in children and its high immunization prevalence, boosting but not replacing or abolishing BCG would be a promising TB vaccination strategy for adults. However, researches have shown that repetitive BCG vaccinations would not enhance the protective efficacy but result in pathological damage in guinea pigs, neonatal calves and mice (3–5). Thus, there is an ongoing quest for a novel TB vaccine that may be used as a BCG supplementary vaccine.

CD8^+^ T cells play a critical role in controlling and eliminating *Mtb*. But unfortunately, BCG is unable to induce efficient CD8^+^ T cell responses (6, 7), and this might be part of the reasons for its unreliable protection efficacy. An effective TB vaccine should be able to incite robust CD8^+^ T cell immunity. *Listeria monocytogenes* (LM) and *Listeria ivanovii* (LI) are facultative intracellular bacteria that share similar intracellular life cycle and virulence determinants (8). They are capable of entering to both phagocytic and non-phagocytic cells, escaping from the phagocytic vacuole, surviving, and multiplying in the host cell cytoplasm and directly spreading to adjacent cells, presenting target antigens, which thereby elicit robust T cell-mediated immune responses, especially CD8^+^ T cell responses (8, 9). A special virulence gene cluster, LIPI-1, containing the genes *prfA, plcA, hly, actA, plcB*, and *mpl* genes is responsible for key steps in this intracellular parasitic life cycle (8). An LM strain or an LI strain deficient in *actA* and *plcB* (*L. monocytogenes* Δ*actA*Δ*plcB* or *L. ivanovii* Δ*actA*Δ*plcB*) is less virulent than the parent strain but induces innate immune response and prime strong T-cell immune responses (9, 10). TB vaccine candidates based on attenuated LM and LI vectors for delivering *Mtb* antigens have been constructed and indicated promising effect on inciting CD8^+^ immunity (10–16).

Antigenic genes are also believed to play an essential role in developing vaccine candidates. While some novel TB vaccine candidates in clinical trials induced strong CD8^+^ T cell responses, most of them were constructed with antigenic genes related to the early stage of *Mtb* infection (17). Indeed, several scientists have confirmed that recombinant BCG strain containing both early-stage and latency-stage associated antigens was more effective (18, 19). Moreover, the multistage vaccine (Ag85B-ESAT-6-*Rv2660c*) was constructed and used to protect against not only active tuberculosis but also reaction of LTBI (19). Assumed a multistage antigen will provide better immunogenicity and protection against *Mtb* infection, we explored the *Listeria*-vectored vaccines expression of multiple *Mtb* antigens, including highly immunostimulatory secreted antigen absent from BCG.

In this study, two attenuated *Listeria* recombinant strains, *L. monocytogenes* Δ*actA*Δ*plcB*-*msv* (LMΔ*-msv*) and *L. ivanovii* Δ*actA*Δ*plcB*-*msv* (LIΔ*-msv*), containing fusion T cell epitopes of 4 *Mtb* antigens (*Rv2460c, Rv2660c, Rv3875*, and *Rv3804c*) were constructed. Among them, *Rv2460c* was used for the first time in TB vaccine construction. *Rv2460c* is upregulated in bacilli reactivation (20). So it is regarded as a marker of TB reactivation. *Rv2660c* is highly upregulated in the latent stage of *Mtb* infection, and recent data have shown it could elicit strong protective responses (21), suggesting that *Rv2660c* might be a promising candidate target for controlling latent tuberculosis infection. We found that intravenous vaccination with either LMΔ*-msv* or LIΔ*-msv* induced specific CD4^+^ and CD8^+^ T cell responses. Furthermore, the recombinant *Listeria* boost strategy was capable of eliciting stronger CD8^+^ T cell response as well as better protection compared with BCG immunization alone.

## Materials and Methods

### Mice

Specific pathogen-free (SPF) female C57BL/6J mice aged 6–8 weeks (Dashuo, China) were housed in a controlled environment (12 h daylight cycle, temperature of 23 ± 2°C and humidity of 50–60%) with food and water *ad libitum* in the animal facility of the Animal Center at the School of Public Heath, Sichuan University. All mouse experiments were performed in compliance with the guidelines of the Animal Care and Use Committee of Sichuan University and approved by the committee.

### Construction of the Recombinant Strains

Plasmids and bacteria strains utilized in this study are presented in Table 1. Potential MHC-I, MHC-II binding T-cell epitopes of C57BL/6J mice (H-2K^b^) in clpP2 (encoded by *Rv2460c*), a hypothetical protein (encoded by *Rv2660c*), ESAT-6 (encoded by *Rv3875*), and Ag85A (encoded by *Rv3804c*) were predicted by using epitope prediction softwares online (http://www.syfpeithi.de/, http://www.cbs.dtu.dk/services/NetMHCpan/, and http://www.imtech.res.in/raghava/propred/). Epitopes of the four genes were tandem-fused through linker (GCCGCCTAC) and ligated into pUC57 vector, resulting in pUC57-*msv* (Figure S1).

**Table 1:** Plasmids and bacterial strains used in this study.

**Plasmid or strain**	**Description**	**Source (References)**
pCW203	Amp^R^, containing the hemagglutinin (HA) epitopes between *Bam*H I and *Hin*d III	Our laboratory [Wang C. et al. (22)]
pUC57-*msv*	Amp^R^, containing a fused antigenic gene of *M. tuberculosis* between *Hin*d III and *Xho* I	This study (Provided by GenScript Biotech corp)
pCW154	Amp^R^ and Ery^R^, containing fragment *L. ivanovii mpl*-*Bam*H I- *Xho* I- *L. ivanovii* orf*BAldh*	Our laboratory [Wang C. et al. (22)]
pCW702	Derived from pCW154, containing fragment *L. monocytogenes mpl*-*Bam*H I- *Xho* I- *L. monocytogenes* orf*BAldh*	Our laboratory [Mahdy et al. (16)]
pCW203-*msv*	Derived from pCW203, containing *msv* antigenic gene	This study
pCW154-*msv*	Derived from pCW154, containing antigen expression cassette with *msv* gene	This study
pCW702-*msv*	Derived from pCW702, containing antigen expression cassette with *msv* gene	This study
PET-*Rv0129c*	Amp^R^, containing the *Rv0129c* gene of BCG	This study
LMΔ*-lacZ*	*L. monocytogenes* Δ*atcA*Δ*plcB-lacZ, actA, plcB* deletion and *lacZ* expression mutant of wild-type *L. monocytogene* strain 10403s	Our laboratory [Mahdy et al. (16)]
LIΔ*-lacZ*	*L. ivanovii* Δ*atc*Δ*AplcB-lacZ, actA, plcB* deletion and *lacZ* expression mutant of wild-type *L. ivanovii* strain PAM55	Our laboratory [Wang C. et al. (22)]
LMΔ*-msv*	Derived from *L. monocytogenes* Δ*atcAplcB-lacZ*, expressing *msv*	This study
LIΔ-*msv*	Derived from *L. ivanovii* Δ*atcAplcB-lacZ*, expressing *msv*	This study

pCW203-*msv* was constructed by cloning into pCW203 a 1028-bp *msv* fragment from pUC57-*msv*. The *Bam*HI- *Xho*I fragment from pCW203-*msv* containing the hemagglutinin (HA) epitopes (TPTAVPATA) and *msv* gene was then ligated into pCW702 or pCW154 (Figure S2). The targeting plasmids pCW702-*msv* and pCW154-*msv* were introduced into the attenuated *Listeria* strain *L. monocytogenes* Δ*actA*Δ*plcB*-*lacZ* (LMΔ*-lacZ*) and *L. ivanovii*Δ*actA*Δ*plcB*-*lacZ* (LIΔ*-lacZ*), respectively. And then erythromycin-resistant and blue colonies were selected. Integration and allelic exchange were performed as previously described (10). Briefly, the blue colonies were selected at 42°C on BHI agar plates supplemented with erythromycin (3 μg/mL) to select for single-chromosomal-crossover constructs (blue). Putative constructs were subsequently grown in BHI broth for six consecutive passages without antibiotic selection at 30°C, the double-chromosomal-cross constructs (white) were diluted and plated on IPTG^+^ X-gal^+^ BHI agar plates. The desired recombinants were identified as erythromycin-sensitive and white. Strains were verified by PCR analysis and sequencing.

### Growth Kinetics Measurement in BHI Broth

Overnight culture of LMΔ*-msv* and LIΔ*-msv* was diluted to OD_600_ of ~0.08 using BHI broth. The diluted bacterial culture was then incubated at 37°C at 180 r/min. Four mL of culture was taken at hourly intervals and the absorbance at 600 nm was measured. The experiment was done in triplicate.

### Heterologous Protein Expression by Two Recombinant Strains in Broth Culture and in Mouse Macrophage-Like Cells

To assess the heterologous protein expression by the recombinant strains in broth, we collected total intracellular and extracellular proteins by trichloroacetic acid precipitation (10).

To assess the heterologous protein expression by both recombinant strains within mouse macrophage-like cells, we infected monolayers of RAW264.7 cells with *Listeria* strains at a MOI of 100:1 for 1 h. Cells were washed and treated with DMEM containing 200 μg/mL gentamicin for 1 h to kill the extracellular bacteria, and further incubated in DMEM medium containing 20 μg/mL gentamicin for 6 h before washing with PBS and lysing cells with RIPA Buffer (Solarbio, China). Total proteins were collected.

Proteins were separated on an 8% SDS gel, transferred to a PVDF membrane, incubated with HA monoclonal antibody (1:5,000) (Sigma-Aldrich, USA). Following the step of horseradish peroxidase (HRP) conjugated anti-mouse secondary antibody incubation (1:1,000) (Beyotime Institute of Biotechnology, China), the signals were developed using Super Signal West Pico (Thermo Scientific, USA).

### *Msv* mRNA Transcription Levels of Two Recombinant Strains in Broth Culture and in Mouse Macrophage-Like Cells

RAW264.7 cells were grown at 37°C with 5% CO_2_ for 24 h in 12-well plates and infected with *Listeria* strains at a MOI of 100:1 for 1 h. Cells were washed and treated with DMEM containing 200 μg/mL gentamicin for 1 h to kill the extracellular bacteria, and then were kept incubated in medium containing 20 μg/mL gentamicin for additional 5 h.

Total RNA of bacteria from BHI broth or RAW264.7 cells was isolated using a RNAprep Bacteria Kit (TianGen, China), and was reverse transcripted to cDNA using the iScript™ gDNA Clear cDNA Synthesis Kit (Bio-Rad, USA). *msv* mRNA transcript abundance was normalized to *rpoB* mRNA transcript abundance. *msv* was amplified by primers *msv-f* : 5′-TGATCGTGTACGTGGAGCAG-3′ and *msv-r*: 5′-GGACCACCACTCATAGCACC-3′, LM- *rpoB* was amplified by primers LM- *rpoB-f* : 5′-AATCGGGGACAATGACT-3′ and LM- *rpoB-r*: 5′-GTGTGCGGAAACCTAC-3′, LI- *rpoB* was amplified with primers LI- *rpoB-f* : 5′-TCCGTTCAGAAAACTTAGCGGT-3′ and LI- *rpoB-r*: 5′-GCAGTTACAGCAGCACCAGAGT-3′. Quantification was performed on the CFX96 (Bio-Rad) with an initial 98°C for 2 min, followed by 40 cycles of denaturation at 98°C for 5 s, annealing and extension at 55°C for 5 s. Melting point analysis was performed to ensure the specificity of the amplification.

### Virulence of the Recombinant Strains in Mice

Virulence of each recombinant strain in mice was detected as previously described Mahdy et al. (16). In brief, three groups of mice (7 mice/group) were injected intravenously (i.v.) with increasing dose of LMΔ*-msv* or LIΔ*-msv* suspended in normal saline. The median lethal dose (LD_50_) values were calculated using the improved Karber method.

### Bacterial Load and Histopathological Analysis in Organs of Infected Mice

Fifty-four mice were randomly distributed into two infection groups and one naïve group (18 mice in each group). The mice were immunized i.v. with LMΔ*-msv* (0.1 × LD_50_), LIΔ*-msv* (0.1 × LD_50_), or normal saline, respectively. For detecting bacterial load, three mice of each group were sacrificed at 1, 2, 3, 5, 7, and 14 dpi, respectively. Samples of liver and spleen tissue were taken aseptically, homogenized with normal saline and plated onto BHI plates. For histopathological analysis, samples of liver and spleen tissue were fixed in 4% paraformaldehyde. Five micrometers of paraffin-embedded sections were cut and stained with hematoxylin-eosin. Histological lesions were examined under 100 × magnification. The experiment was done in triplicate.

### Intracellular Cytokine Staining

Sixty-three mice were randomly divided into nine groups (seven mice in each). Five groups were immunized i.v. with LMΔ-*msv*, LIΔ-*msv*, LMΔ-*lacZ*, LIΔ-*lacZ*, and NS. The other four groups were prime-boost immunized i.v. with LMΔ-*msv* → LMΔ-*msv*, LMΔ-*msv* → LIΔ-*msv*, LIΔ-*msv* → LMΔ-*msv*, and LIΔ-*msv* → LIΔ-*msv*, respectively. Boost immunization was carried out 40 days after prime immunization. Immunization doses were 0.1-fold of the 50% lethal doses of each strain. Splenocytes were harvested from the immunized mice 9 days after the last immunization. Isolated splenocytes were stimulated with the peptide pool listed in Table 2 at the final concentration of 1 μg/mL prepared in 10 μg/mL Golgi stop (BD PharMingenTM, USA) or no stimulant in 96-well plates at 37°C for 5 h, and then processed for flow cytometry analysis as described previously (16). After centrifuging for 5 min at 1, 300 × g, the cells were then washed and co-stained with FITC-Anti-Mouse CD3 (BD, USA), PerCP-Anti-Mouse CD4 (BD, USA), and APC-CY7-Anti-Mouse CD8 (BD, USA) at 4°C for 30 min. The surface-stained cells were washed and permeabilized using Cytofix/Cytoperm kit (BD, USA). The cells were then washed and stained for 45 min on 4°C for PE-Anti-Mouse IFN-γ (Biolegend, USA), PE-Cy7-Anti-Mouse TNF-α (Biolegend, USA), and APC-Anti-Mouse IL-2 (Biolegend, USA). The stained cells were analyzed using flojow 10.0.

**Table 2:** Peptides used in this study.

**No**	**Peptide sequence**	**Protein origin and position**
1	IQPQARYILPSFI	ClpP2_8−20_
2	KESNPYNKLFEERIIFLGVQV	ClpP2_28−48_
3	NDIMAQLLVLESLDPDRDITMY	ClpP2_52−75_
4	SPGGGFTSLMAIYDTMQYVRADIQTVCL	ClpP2_78−105_
5	AEIERMRTL	ClpP2_155−163_
6	IRKDTDRDK	ClpP2_177−187_
7	AEEAKDYGIIDTVLEYRKL	ClpP2_191−209_
8	SPDFVDETAGQSWCAILGLNQF	Hypothetical protein (*Rv2660c*)_51−69_
9	TEQQWNFAGIEAAASAIQ	ESAT-6_2−19_
10	TFLTSELPGWLQANRHVKPT	Ag85A_142−161_
12	YAGAMSGL	Ag85A_189−196_
13	PTLIGLAMGDAGGY	Ag85A_205−218_
14	WEYWGAQLNA	Ag85A_276−287_

### Antigen-Specific IgG Assay

Twenty-five mice were randomly divided into five groups (five mice in each). Five groups were immunized i.v. with LMΔ-*msv*, LIΔ-*msv*, LMΔ-*lacZ*, LIΔ-*lacZ*, or NS. Two weeks after the primary immunization, mice were immunized with the same strain for a booster dose. Two weeks after the last immunization, serums were collected and total IgG titers of anti-msv proteins were detected using ELISA. ELISA plates were coated overnight at 4°C with 100 μL msv protein (5 μg/mL) in NaHCO_3_ buffer (pH 9.6). The washed plates were then blocked with bovine serum albumin 1% (w/v) in washing buffer for 2 h at 37°C. Diluted serum (1:100) were added and incubated at 37°C for 1 h. Horseradish peroxidase labeled goat anti-mouse IgG (1:250) (Beyotime, China) was added and incubated at 37°C for 30 min. The TMB substrate was added into the washed plates, and the reaction was stopped 20 min later. The antibody levels were expressed in OD_450nm_ values.

### Protection Against BCG Challenge in Mice

One hundred mice were randomly distributed into five groups, NS → NS → NS, BCG → NS → NS, NS → LMΔ-*msv* → LIΔ-*msv*, and BCG → LMΔ-*msv* → LIΔ-*msv*. The mice were either immunized subcutaneously with 1 × 10^6^ CFU of BCG or normal saline around the hind legs, and then immunized i.v. at the dose of 0.1-fold of LD_50_ of each strain on 14 and 54 dpi. Nine days after the last immunization, splenocytes were harvested from the immunized mice (10 mice/group), and the intracellular cytokine staining assay was performed as described previously (16). Cells were stained with FITC-Anti-Mouse CD3 (BD, USA), PerCP-Anti-Mouse CD4 (BD, USA), APC-CY7-Anti-Mouse CD8 (BD, USA), PE-Anti-Mouse IFN-γ (Biolegend, USA), PE-Cy7-Anti-Mouse TNF-α (Biolegend, USA), and APC-Anti-Mouse IL-2 (Biolegend, USA). The stained cells were analyzed using flojow 10.0.

Four weeks after the final immunization, the mice were intranasally challenged with 1 × 10^7^ CFU of *Mycobacterium bovis* BCG. Three weeks after challenge, the mice were sacrificed, and the serum, the spleen and the lung were collected. The lung and spleen were homogenized in PBS. Then the total genomic DNA was isolated from lung or spleen homogenate by using a TIANamp Bacteria DNA Kit (TianGen, China), and used as the template for the next q-PCR. Then the mycobacterial burden was determined by q-PCR, using *Rv0129c* specific primers (forward primer 5′-CAACGACCCAATGGTTCAGAT-3′, reverse primer 5′-CAGGAACTTCGCCGGTATGT-3′), and specific probe (5′-FAM-CAACAACACCCGGATCTGGGTGTAC-3′-BHQ). q-PCR was performed with SGExel Goldstar Taqman Mixture (Sangon Biotech, China) on the CFX96™ Real-Time system (Bio-Rad, USA) according to the manufacturer's instruction. A standard curve was obtained as follow. First, extracted genomic DNA from 10-fold serial dilution of BCG suspensions and CFU of the same BCG aliquots were quantified by culturing on solid medium. Bacterial genomic DNA was extracted from each 1 mL of diluted suspension, and was eluted in a final volume of 50 μL. Two μL of each 50 μL DNA sample was subsequently used for each PCR reaction. The standard curve was obtained by the Ct (cycle threshold) values as Y axis and the Log (CFU) of BCG as X axis. The PCR limits of CFU detection in a 1 ml tissue homogenate therefore ranged from102.45 CFU to 108.45 CFU (*R*^2^ = 0.999) (Figure S3). Results are expressed as average of three independent reactions. The left lung of each mice was excised and examined for histological lesions as described before (15). Briefly, Lung sections were stained with hematoxylin-eosin. Ten different fields within each slide were imaged and evaluated by two pathologists who were not aware of sample assignment to experimental groups. The levels of SP-A in the serum or lung and ADA in the lung was detected by ELISA (Shanghai Enzyme-linked Biotechnology).

### Statistical Analysis

One-way or two-way analysis of variance (ANOVA) with Tukey's multiple-comparison test was performed by using SPSS 20.0 (IBM SPSS 20.0, USA) to determine significance in comparisons of mean frequencies of cytokine-producing CD4 and CD8 T cells among mice in the vaccinated and control groups. All results were considered statistically significant at *P* < 0.05.

## Results

### Construction and Characterization of *Listeria* Recombinant Strains

**Figure 1:**
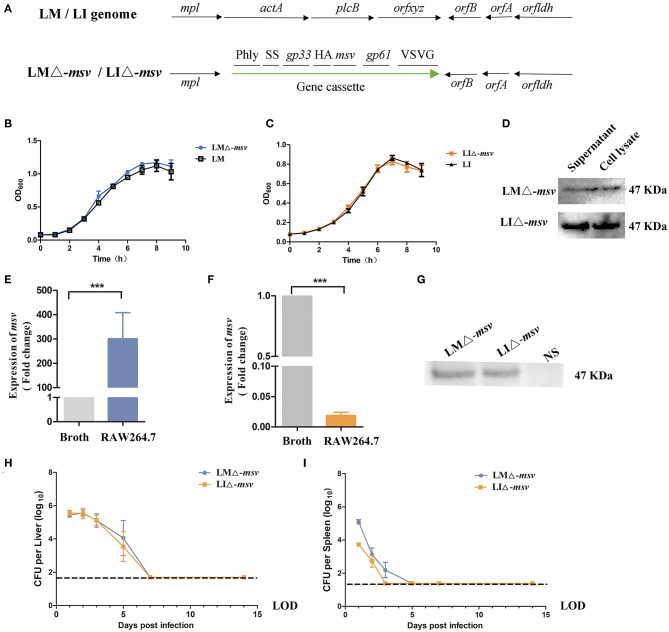
Characterization of the recombinant strains LMΔ-*msv* and LIΔ-*msv*. **(A)** The genome of wild-type and recombinant *Listeria* strains. The antigen cassette was integrated into LM or LI genome. It included the following seven components: (□) Phly is the promoter of *hly* from LM; (□) SS is the secretory signal from the LM *hly* gene; (□) gp33 is the epitopes (KAVYNFATM) from *LCMV* for stimulation of CD8^+^ T cells; (□) HA is the human influenza hemagglutinin epitopes (YPYDVPDYA) as a Western blot marker; (□) *msv* is the fusion antigen of *Mtb*; (□) gp61 is the epitopes (GLKGPDIYKGVYQFKSVRFD) from *LCMV* for stimulation of CD4^+^ T cells; (□) VSVG is a epitopes (YTDIEMNRLGK) from *vesicular stomatitis* virus as a Western blot marker. **(B,C)** The *in vitro* growth curve of LMΔ-*msv* (■) and LM wild-type strain (□) or LIΔ-*msv* (•) and LI wild-type strain (▴). **(D)** Western blotting of culture supernatant and cell lysate from LMΔ-*msv* and LIΔ-*msv* using the anti-HA monoclonal antibody as primary antibody. **(E,F)** Transcription levels of *msv* in BHI broth or in RAW264.7 infected with LMΔ-*msv* or LIΔ-*msv* using real-time qPCR. RAW264.7 cells were infected at a MOI of 100:1 with LMΔ-*msv* or LIΔ-*msv*. At 6 h post-infection, the infected cells were lysed. Total RNA of bacteria that harvested from BHI broth and RAW264.7 cells, respectively. *msv* mRNA transcript abundance was normalized to *rpoB* mRNA transcript abundance. The bacterial *msv* mRNA transcription levels in the infected RAW246.7 cells were compared with those in broth. **(G)** Western blot of cell lysates using an anti-HA monoclonal antibody to detect the fusion msv protein. RAW264.7 cells were uninfected or infected at a MOI of 100:1 with LMΔ-*msv* or LIΔ-*msv*. At 7 h post-infection, the infected cells were lysed and subjected to Western blot. **(H,I)** Bacterial loads in liver and spleen after intravenous administration. Mice were intravenous inoculated with 10^6^ CFU of LMΔ-*msv* (■) or 10^7^ CFU of LIΔ-*msv* (•). Livers and spleens were collected at 1, 2, 3, 5, 7, 14 dpi. Results presents as mean ± SEM per group of nine mice. The dotted lines represent the detection limits in each experiment. All the experiments were performed in triplicate. ***P < 0.001 (by one-way ANOVA with Tukey's multiple-comparison test). LOD, limit of detection.

Cellular immunity is crucial for protecting against *Mtb*. To design a novel TB vaccine, protein sequences produced by *Rv2460c, Rv2660c, Rv3875*, and *Rv3804c* genes were predicted for T cell epitopes using epitopes prediction softwares online. Twenty five polypeptides, including ClpP2_8−20_ (IQPQARYILPSFI), ClpP2_28−48_ (KESNPYNKLFEERIIFLGVQV), ClpP2_52−75_ (SANDIMAQLLVLESLDPDRDITMY), ClpP2_78−105_ (SPGGGFTSLMAIYDTMQYVRADIQTVCL), ClpP2_109−117_ (ASAAAVLLA), ClpP2_119−135_ (GTPGKRMALPNARVLIH), ClpP2_155−163_ (AEIERMRTL), ClpP2_177−187_ (IRKDTDRDK), ClpP2_191−209_ (AEEAKDYGIIDTVLEYRKL), hypothetical protein (*Rv2660c*)_41−49_ (VVAPSQFTF), hypothetical protein (*Rv2660c*)_52−69_ (RSPDFVDETAGQSWCAILGLNQF), ESAT-6_14−22_ (AASAIQGNV), ESAT-6_50−76_ (AYQGVQQKWDATATELNNALQNLARTI), ESAT-6_82−90_ (AMASTEGNV), Ag85A_3−23_ (LVDRVRGAVTGMSRRLVVGAV), Ag85A_46−63_ (RPGLPVEYLQVPSPSMGR), Ag85A_73−82_ (GANSPALYLL), Ag85A_87−95_ (AQDDFSGWD), Ag85A_103−121_ (WYDQSGLSVVMPVGGQSSF), Ag85A_140−165_ (WETFLTSELPGWLQANRHVKPTGSAV), Ag85A_188−212_ (VYAGAMSGLLDPSQAMGPTLIGLAM), Ag85A_233−241_ (QRNDPLLNV), Ag85A_243−263_ (KLIANNTRVWVYCGNGKPSDL), Ag85A_265−273_ (GNNLPAKFL), Ag85A_276−287_ (FVRTSNIKFQDA), Ag85A_292−308_ (GGHNGVFDFPDSGTHSW), and Ag85A_318−333_ (MKPDLQRALGATPNTG) were chosen based on their scores. We constructed a tandem-fusion antigen, called *msv*, containing potential T cell epitopes of C57BL/6J mice from clpP2 (encoded by *Rv2460c*), a hypothetical protein (encoded by *Rv2660c*), ESAT-6 (encoded by *Rv3875*), and Ag85A (encoded by *Rv3804c*) (Figure S1). The fusion protein msv could induce significant humoral and cellular immunity responses in C57BL/6J mice (Figure S4). The *msv* gene was coding-optimized for being expressed in LM 10403s and then was introduced at the downstream of the *hly* promoter in antigen expression cassette of targeting plasmid (Figure S2). The antigen expression cassettes containing *msv* were integrated into *L. monocytogenes* Δ*actA* Δ*plcB* or *L. ivanovii* Δ*actA* Δ*plcB* chromosome to construct recombinant strains, LMΔ-*msv* and LIΔ*-msv* (Figure 1A). PCR analysis (Figure S5) and sequencing were used to confirm integration.

As shown in Figures 1B,C, recombinant strains grew as fast as their parental wild-type strains in BHI broth. The expression of the fusion protein was confirmed by western blotting with rabbit anti-HA monoclonal antibody. In Figure 1D, both strains were cultured in BHI broth, and the total protein samples were obtained from bacterial cell lysate as well as culture supernatant. The specific 47 kDa target band appeared in both bacterial cell lysate and supernatant indicated that fusion msv protein was successfully expressed in recombinant LMΔ-*msv* and LIΔ-*msv*, although the protein amount expressed in LMΔ-*msv* was significantly lower. To explore whether the msv protein expression by recombinant *Listeria* strains would be promoted in the cell, RAW264.7 cells were infected with both strains individually for 6 h, and cells lysates were collected. Interestingly, although the mRNA level of *msv* in LMΔ-*msv* infected cells increased by nearly 300 times to that in broth and the mRNA level in LIΔ-*msv* infected cells decreased by 50 times to that in broth (Figures 1E,F), the amount of msv fusion proteins from both infected cells were nearly the same (Figure 1G).

### The Virulence of Recombinant Strains Was Attenuated

To evaluate the virulence of those recombinant vaccines, we determined the LD_50_ value, bacterial growth kinetics *in vivo* and histological changes of target organs in C57BL/6J mice. The LD_50_ value of LMΔ-*msv* was 7.8 × 10^7^ CFU per mouse, and that of LIΔ-*msv* was 2.0 × 10^8^ CFU per mouse, both were two logs higher than those of their parental wild type strain (Figure S6).

The ability of recombinant strains to multiply in the liver and spleen of infected mice was evaluated by detecting the bacterial load in the liver and spleen at different days post-intravenously infecting the mice at the dose of 0.1 × LD_50_. As indicated in Figure 1H, the two recombinant strains possessed a similar growth trend in the liver. The bacterial counts in liver kept increasing for 2 days after infection, reaching 10^5^ CFU bacteria per liver. Then the bacteria were completely eliminated from liver by day 7. The recombinant strains in the spleen were eliminated faster than those in the liver. No bacteria could be detected in spleen by day 3 or day 5. These results suggested that the recombinant strains invaded liver and spleen, lasted for several days, then were completely eliminated.

**Figure 2:**
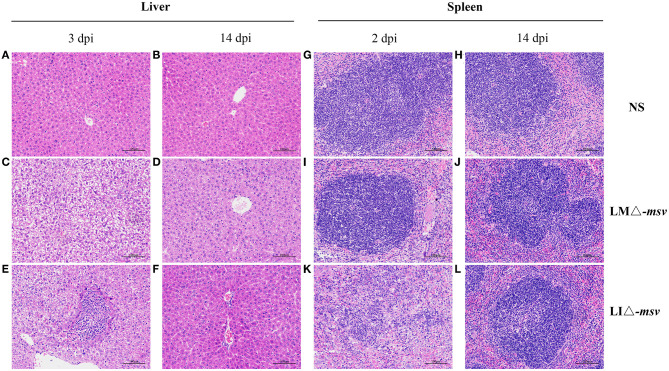
Histological pathology in liver and spleen after intravenous administration. Mice were intravenously inoculated with 10^6^ CFU of LMΔ-*msv*, 10^7^ CFU of LIΔ-*msv* or 100 μl normal saline. Liver **(A-F)** and spleen **(G-L)** tissues were collected at 2, 3, or 14 dpi and were fixed in 4% paraformaldehyde for 24 h. Five micrometers of paraffin-embedded sections were cut and stained with hematoxylin-eosin. Histopathology changes in liver and spleen were observed and imagined under 100× microscope. Representative pathology is indicated by arrows. The images presented are representatives of changes observed in the mice.

Histopathological lesions in the liver and spleen were observed in 0.1 × LD_50_ recombinant strains infected mice (Figure 2). The main histopathological changes in the liver was hepatic necrosis (3 dpi) and in the spleen was lymphocytic hyperplasia (2 dpi). The liver and spleen tissues restored to normal at 14 dpi. Such results were consistent with the bacterial growth curve in organs. With the decrease of the bacterial load, the histopathological damage alleviated and even disappeared. Also, the changes of AST level in serum (Figure S7) confirmed again that immunization with the two recombinant strains only resulted in mild and reversible pathological changes.

### Recombinant Strains Elicited a Robust Immune Responses

**Figure 3:**
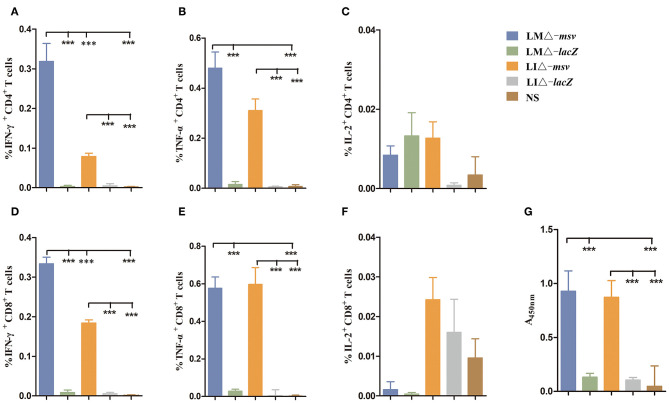
Mice cellular and humoral immune responses induced by recombinant strains. **(A–F)** Flow cytometric analysis for cytokine secreting T cells in the splenocytes of mice. C57BL/6J (seven mice/group) mice were intravenously immunized with LMΔ-*msv*, LIΔ-*msv*, LMΔ-*lacZ*, LIΔ-*lacZ*, and normal saline, respectively. Splenocytes were collected at 9 dpi, stimulated with msv mixed peptides (1 μg/mL) in the presence of Golgistop (10 μg/mL), and analyzed for cytokine production cells by ICS assay. CD3^+^CD4^+^ cells (CD4^+^ T cells) and CD3^+^CD8^+^ cells (CD8^+^ T cells) that produce IFN-γ, TNF-α, and IL-2 were counted. Summary data of single cytokine producing CD4^+^ and CD8^+^ T cells are shown with significant differences indicated. **(G)** Antibody levels in mice serum after immunization with recombinant strains. C57BL/6J (five mice/group) mice were intravenously immunized with LMΔ-*msv*, LIΔ-*msv*, LMΔ-*lacZ*, LIΔ-*lacZ*, and normal saline, respectively. Two weeks after the first immunization, mice were immunized with the same strain for a booster dose. Two weeks after the second immunization, total anti-msv IgG titers in mice serum were detected using ELISA. All the experiments were performed in triplicate. Each bar represents the mean ± SEM for a group of seven mice from one independent experiment. ***P < 0.001 (by one-way ANOVA with Tukey's multiple-comparison test).

Reduction of strain virulence enhances its safety, however on the other hand, this may also weaken its immunogenicity. We compared the cellular and humoral immune responses of recombinant attenuated strains immunized mice with the mice immunized with vector strains or normal saline. Flow cytometric analysis of splenocytes at 9 dpi revealed that the percent of IFN-γ secreting or TNF-α secreting CD4^+^ and CD8^+^ T cells were significantly increased in mice immunized with recombinant attenuated strains (LMΔ-*msv* and LIΔ-*msv*) compared with control groups (*P* < 0.001) (Figures 3A–F). Furthermore, compared to the mice immunized with LIΔ-*msv*, more IFN-γ secreting CD4^+^ and CD8^+^ T cells in mice immunized with LMΔ-*msv* were detected (*P* < 0.001) (Figures 3A,D). In addition, antibody levels in mice serum were evaluated by ELISA using purified msv protein as antigen. As expected, both LMΔ-*msv* and LIΔ-*msv* induced significantly higher IgG levels than controls (Figure 3G).

**Figure 4:**
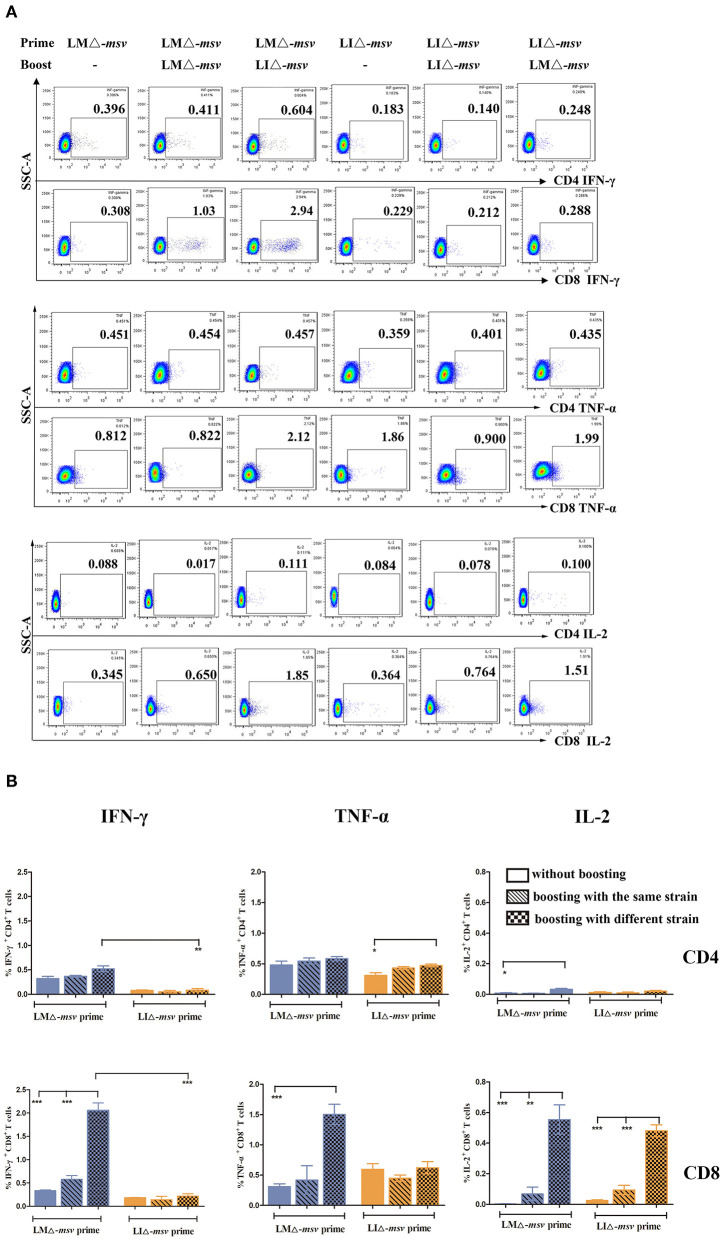
Flow cytometric analysis for cytokine secreting T cells in the splenocytes of mice after boost immunization. Forty days after primary immunization, C57BL/6J mice (seven mice/group) were boosted with the same or different strain. Splenocytes were collected at 9 days after last inoculation, then stimulated for 5 h with msv mixed peptides (1 μg/mL) and analyzed for cytokine production by ICS assay as described in Figure 3. **(A)** Representative flow cytometric plots of intracellular staining in CD4^+^ and CD8^+^ T cells. **(B)** Summarized data of single cytokine producing CD4^+^ and CD8^+^ T cells. All the experiments were performed in triplicate. The data shown in **(A)** is representative and that shown in **(B)** is mean value. *P < 0.05, **P < 0.01, and ***P < 0.001 (by two-way ANOVA with Tukey's multiple-comparison test).

CD8^+^ T cell response is crucial in protecting and controlling *Mtb* infection. To obtain stronger CD8^+^ T cell immune responses, we used prime-boost strategy by boosting the mice 40 days after primary vaccination (Figure S8). As shown in Figure 4, LMΔ-*msv* → LIΔ-*msv* induced the highest percent of CD4^+^ IFN-γ^+^ T, CD8^+^ IFN-γ^+^, CD8^+^ TNF-α^+^, and CD8^+^ IL-2^+^ T cells among all experimental groups. It indicated that prime-boost immunization strategy, especially heterologous prime-boost, that is to say using different strain as secondary immunization vaccine notably enhanced CD8^+^ T cell responses.

### Enhanced T Cell Responses by BCG Prime-Recombinants Boost

**Figure 5:**
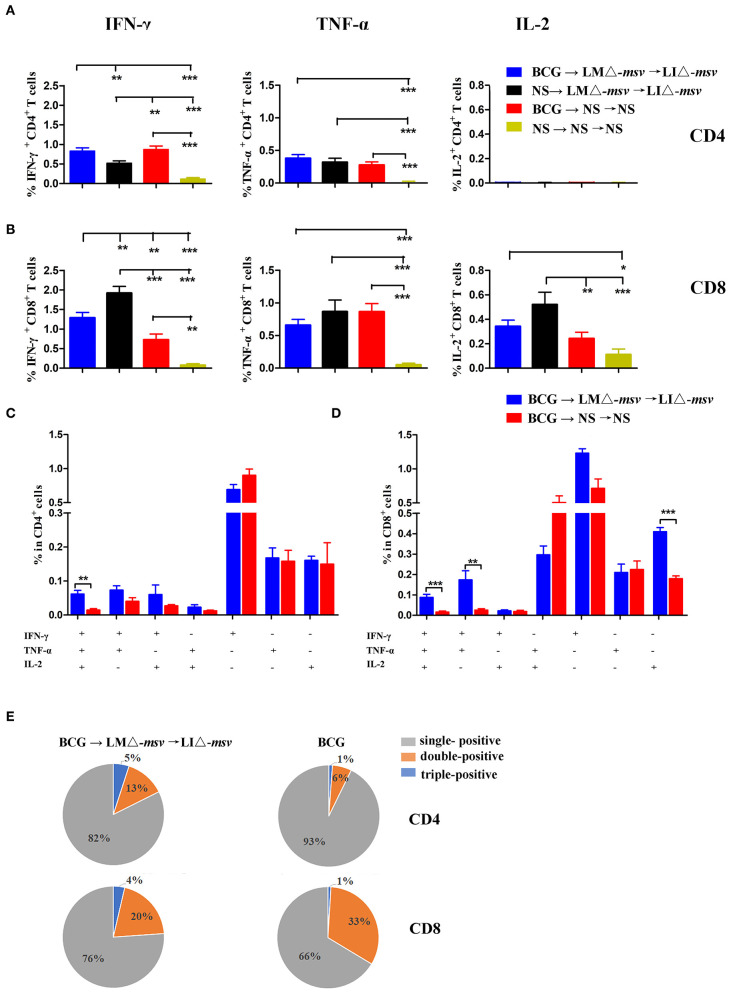
Boosting with LMΔ-*msv* → LIΔ-*msv* increased T cell responses primed by BCG immunization. C57BL/6J (10 mice/group) primed by BCG were boosted with LMΔ-*msv* → LIΔ-*msv* or not. Control mice received normal saline, or immunized with BCG alone or LMΔ-*msv* → LIΔ-*msv*. Splenocytes were collected at the 9th day after the last inoculation, stimulated for 5 h with msv mixed peptides (1 μg/mL), and analyzed for cytokine production by ICS assay as described in Figure 3. **(A,B)** Summarized data of single cytokine producing CD4^+^ and CD8^+^ T cells **(C,D)** Characterization of poly-functional T cell responses in the spleen. The proportions of CD4^+^ or CD8^+^ T cells that produce one, two, or three cytokines are shown in the bar graph **(E)** The proportion of the three types of CD4^+^ and CD8^+^ T cells are shown in the pie chart. All the experiments were performed in triplicate. Each point represents the mean ± SEM for a group of seven mice from one independent experiment. *P < 0.05, **P < 0.01, and ***P < 0.001 (by two-way ANOVA with Tukey's multiple-comparison test, the *P*-value for pairwise mean comparison were adjusted for multiple comparison under this model by using the Bonferroni correction).

To further assess whether the LMΔ-*msv* → LIΔ-*msv* immunization strategy could enhance T cell responses after BCG immunization, we vaccinated the mice with BCG, LMΔ*-msv* and LIΔ*-msv* sequentially (Figure S9). Nine days after the last immunization, splenocytes were collected from different groups and stimulated with the msv peptide pool or incubated with medium as control. Percents of IFN-γ, TNF-α, and IL-2 positive T cells were compared between groups (Figures 5A,B). In the spleen, LMΔ-*msv* → LIΔ-*msv* boost dominantly increased IFN-γ responses in CD8^+^ T cells. The polyfunctional T cells were further analyzed and showed that the IFN-γ^+^TNF-α^+^IL-2^+^-secreting CD4^+^ T cells were significantly boosted (Figures 5C,E), and the IFN-γ^+^TNF-α^+^IL-2^+^, IFN-γ^+^TNF-α^+^, and IL-2^+^ secreting CD8^+^ T cells were boosted too (Figures 5D,E). In addition, PPD-specific CD4^+^ T and CD8^+^ T cell responses in spleen were also boosted (Figure S10).

### Attenuated Recombinant *Listeria* Strains Boost Enhanced BCG-Induced Protection

**Figure 6:**
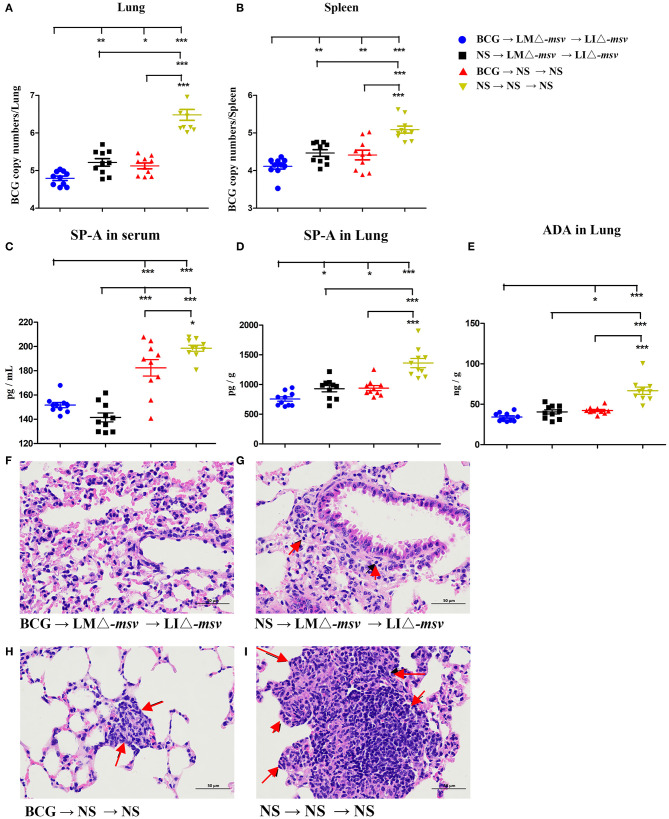
Protective efficacy against BCG challenge. Four weeks after the last immunization, mice (10 mice/group) were intranasally infected with 1 × 10^7^ CFU of BCG. Three weeks after infection, the mice were sacrificed. **(A,B)** The bacterial numbers in the lungs **(A)** and spleens **(B)** were determined by q-PCR. **(C–E)** SP-A and ADA levels in the serum and lung after the BCG challenge. The SP-A levels in serum **(C)** and lung **(D)** and the ADA levels in lung **(E)** were determined using ELISA. **(F–I)** Representative histological appearance of lung tissue sections (H&E staining). Histopathology changes in the lung (left) were observed and imagined under 400× microscope. Representative pathology is indicated by arrows. Ten different fields within each slide were imaged and evaluated by two pathologist who were not aware of sample assignment to experimental groups, and at least 60% have similar histopathological changes. All the experiments were performed in triplicate. Each point represents the mean ± SEM for a group of seven mice from one independent experiment. *P < 0.05, **P < 0.01, and ***P < 0.001 (by two-way ANOVA with Tukey's multiple-comparison test, the *P*-value for pairwise mean comparison were adjusted for multiple comparison under this model by using the Bonferroni correction).

To evaluate the protection against *Mycobacterium* infection, we carried out the mice experiments (Figure S9). Four weeks after the last immunization, C57BL/6J mice were intranasally challenged with 1 × 10^7^ CFU of BCG. We intranasally challenged the mice with *M. bovis* BCG and collected the lung 1 h later. The bacterial burden in the lung was detected by plating serial dilution of tissue homogenates onto Middlebrook 7H10agar plates. The mean retention of BCG in the lung is 75%. Bacterial burden determination and lung histopathological observation were performed 3 weeks after the challenge. The bacterial load in the lung and spleen were both highest in the NS control group (Figure 6) as expected. Importantly, in comparison to immunization with either only *Listeria* recombinant strains or BCG alone, combination with LMΔ-*msv*, LIΔ-*msv*, and BCG resulted in a largest reduction of CFU in the lung (Figure 6A) and spleen (Figure 6B). This group also had the lowest level of SP-A in serum (Figure 6C) and lung (Figure 6D), and the lowest level of ADA in the lung (Figure 6E) at 3 weeks post-challenge. Additionally, the mice in this group showed the lightest pulmonary granulomatous consolidate at 3 weeks post-challenge (Figures 6F–I).

## Discussion

It is generally acknowledged that BCG hardly protects against TB in adults and new vaccination strategies against tuberculosis are needed to enhance the immunity of adolescents and adults. Considering the high coverage of BCG vaccination worldwide (23), a novel vaccine that could be used as the BCG booster would be better. For decades, many different TB vaccines have been implemented, including subunit vaccines and live vector-based vaccines (24), but these vaccines have their own drawbacks, including high costs of purifying protein, a special adjuvant to promote immune responses, and pre-existing immunity to vaccine vectors (24). The failure of an TB vaccine candidate MVA85A that based on a modified Ankara virus vector indicated the need for further study to develop new booster vaccines against TB (2). Attenuated LM has been developed as a vaccine vector for TB vaccine candidates (11–14). Recently, we constructed two attenuated LI recombinant strains expressing Ag85A or ESAT-6, which induced potent CD4^+^ and CD8^+^ T cell responses either in the spleen after intravenous immunization (10) or in the lung after intranasal vaccination (15). Therefore, the facultative intracellular *Listeria* strain is considered as a promising delivery system since it induces strong CD4^+^ and CD8^+^ cell-mediated immune response (9–11). Besides, unlike virus-vectored TB vaccines such as MAV85A, human pre-existing immunity to *Listeria* was lower. This guarantees the immune protection efficacy of the vaccine (25, 26). Therefore, in this study, we prompted a novel BCG booster strategy, which consisted of attenuated recombinant LM and LI.

Potent antigenic genes are crucial for vaccine construction. Current TB vaccine candidates in clinical trials are mostly prophylactic vaccines that target antigens expressed during the early stage of *Mtb* infection. However, the early stage related antigens will be down-regulated in the late infection stage and are therefore insufficient for long-term protection (23, 24). In this study, a fusion antigen gene including T-cell epitopes of four *Mtb* antigenic genes (*Rv2460c, Rv2660c, Rv3875*, and *Rv3804c*) was designed (Figure S1) and a 47-kDa fusion protein was expressed by *Listeria* vector under the control of the *hly* promoter (Figure 1D). Aagaard et al. (19) and Lin et al. (27) confirmed that the multiple *Mtb* antigens combining the early and late stage *Mtb* antigens would be better than a single *Mtb* protein, as the multiple *Mtb* antigens were not only capable of preventing *Mtb* infection but also able to control late-stage infection and reactivation. Other studies also indicated that the protective immunity of antigen proteins fusion of different infectious stage related antigens were better than the individual antigen (28–30). However, whether multistage-fusion epitopes have the same effect is worth exploring further. Ag85A (*Rv3804c*) is a major protein secreted by *Mtb* and presented in the early culture supernatant in broth, which could induce humoral and cell-mediated immunity and were used in most vaccine candidates currently in clinical trials (19, 27, 31, 32). The *Rv2660c* gene is preferentially induced under starvation conditions, which is associated with higher human CD4^+^ T cell response in LTBI people (33). The *Rv2460c* gene is an important participant in *Mtb* growth, which is up-regulated during reaction or under stress conditions (20). To our knowledge, this was the first work regarding recombinant *Listeria*-vectored vaccine candidates that expressed a fusion protein (msv) consisting of the genes related to different TB infectious stages. The expression of Msv was promoted by LM hly promoter (Phly) (Figure 1A) and was regulated by PrfA. Our results showed that the msv expression level of the two recombinants were similar in infected RAW264.7 cells (Figure 1G), but in broth, the extracellular msv protein level of LIΔ-*msv* was higher than that of LMΔ-*msv* (Figure 1D). Previous studies have observed the higher expression of PrfA-dependent proteins in LM during intracellular growth compared to that in broth medium (34). A similar phenomenon was observed in our study (Figure 1E), the mRNA level of *msv* in LMΔ-*msv* infected cells was about 300-fold higher than that in broth, we also found that the mRNA level of *msv* in LIΔ-*msv* infected cells decreased by 50 times to that in broth (Figure 1F). The differences in the activity of PrfA(LM) and PrfA(LI) *in vivo* may explain this, even though there was no difference in their binding affinities and strengths to promote transcription *in vitro* (35).

As an important vaccine delivery, attenuated LM vaccine strains have been confirmed to be safe in humans (36–39), and LI is even safer than LM due to its notably decreased virulence and it only infects ruminants (40). For ensuring a safe application, LM and LI vectors used in this study were attenuated with *actA* and *plcB* gene to result in a 100-fold increase of LD_50_ compared to the wild type strain by intravenous injection (Figure S5). Besides, the histological damage in spleen and liver were mild and recovered to normal at 5 or 7 dpi (Figures 1H,I, 2).

It is generally accepted that CD4^+^ Th1-typed response is important to protect against *Mtb* infection, but CD8^+^ T cell immunity also plays an essential role in immune protection against *Mtb* (9, 32). However, BCG is unable to induce effective CD8^+^ T cell response (6, 9), it might be one of the factors accounting for the limited efficacy of BCG. To evaluate the immunogenicity of the two *Listeria*-based TB vaccine candidates, in this study, we assayed T cell responses specific to msv peptide pool or protein. We found both candidates incited higher percentages of IFN-γ or TNF-α-secreting-CD4^+^ and CD8^+^ T cells in comparison with vector control or NS (Figure 3). It is believed that IFN-γ and TNF-α play important roles in the protective response against *Mtb* by limiting bacilli to multiply and spread (41). LMΔ-*msv* induced stronger cell-mediated responses than LIΔ-*msv*. This may partly be associated with LMΔ-*msv*'s stronger invasive ability and longer survival time in the spleen (Figures 1H,I), and it could be attributed to the difference between listeriolysin O (LLO) and ivanolysin (ILO). A research suggested that the weak ability of LI to colonize in spleen may at least partly be related to that ILO does not possess some properties that belong to LLO (42).

To compare the immune responses at different inoculation strategies, C57BL/6J mice were intravenously immunized with LMΔ-*msv* or LIΔ-*msv*, and 40 days later, they were boosted with the same strain or another strain. After the 2nd vaccination, the strongest increase in the level of spleen cytokine-producing T cells was observed in mice that were prime-boosted with LMΔ-*msv* and LIΔ-*msv*, respectively. Previous studies showed that the negative impact of pre-existing immunity against LM was so low that repeatedly immunized with LM-based vaccines was a promising regimen in prevention of many diseases, including tuberculosis (25, 26). Several studies reported that repeated immunization with the same attenuated LM-based TB vaccine candidate could induce potent antigen-specific T cell responses by intraperitoneal (14), intracutaneous (12) vaccination as well as intravenous (13) inoculation. Similar to these results, Jiang et al. (15) found that mice vaccinated intranasally with the same LI-vectored TB vaccine candidate induced promising lung-localized cellular and humoral immune responses. As shown in Figure 4, our results also confirmed that repeated immunization with the same strain was more effective than single-dose immunization. But interestingly, we also observed that mice prime-boosted with both LMΔ-*msv* and LIΔ-*msv* induced stronger T-cell-mediated immune responses than those immunized with LMΔ-*msv* or LIΔ-*msv* repeatedly. We think the anti-vector immunity of *Listeria* strains does occur, even though they were very low (25). So using different *Listeria* strains for prime and boost will largely decrease the anti-vector immunity, due to many genes or proteins of LM and LI are not completely homologous, although they carry similar virulence gene clusters. To our knowledge, this is the first study to develop a novel vector platform consisting of both attenuated LM and LI in TB vaccine.

Figure 4 indicated that LMΔ-*msv-*prime-LIΔ-*msv-*boost was the most immunogenic vaccination strategy. So, we afterward evaluated its feasibility as a booster vaccine strategy for BCG-primed mice. We found that the antigen-specific CD4^+^ and CD8^+^ T cells were both enhanced after boosting. The proportion of IFN-γ^+^TNF-α^+^IL-2^+^-secreting CD4^+^ T cells and IFN-γ^+^TNF-α^+^IL-2^+^, IFN-γ^+^TNF-α^+^, and single IL-2^+^-secreting CD8^+^ T cells were all increased. Previous studies had shown that polyfunctional T cells were functionally superior to their single-positive counterparts, as such polyfunctional T cells were less differentiated and more long-lived (19, 29, 32). The promising multistage TB vaccine candidate H56 could evoke IL-2^+^IFN-γ^+^TNF-α^+^ and IL-2^+^IFN-γ^+^-secreting CD4^+^ T cells so to provide protection against *Mtb* infection (19). However, some researchers believe that only CD4^+^ T cell responses is insufficient for protection. The famous TB vaccine candidate MVA85 may be attributed to insufficient CD8^+^ T cell responses when given as a booster to previous BCG vaccination (2). Additionally, Caccamo et al. (43) found that polyfunctional CD8^+^ T cells, like IL-2^+^IFN-γ^+^-secreting CD8^+^ T cells were associated with protection against developing TB disease. Commandeur et al. (44) also found that IFN-γ^+^TNF-α^+^ CD8^+^ T cells were the most prominent subsets in long-term latently infected individuals. Therefore, the polyfunctional CD8^+^ T cells play a significant role in TB protection, although its mechanisms require more detailed studies. The notable induction of polyfunctional CD8^+^ T cell responses in this study suggests a promising immunogenicity of such *Listeria*-based *Mtb* delivery system to be a booster of BCG. We are not the first to use *Listeria*-vector TB vaccine as a booster vaccine for BCG, but few of the others analyzed the quantity of the polyfunctional T cell responses in mice or other animal models (11, 12). Yin et al. (11) reported an attenuates LM-vector Tb candidate expressing *Mtb* FbpB and ESAT-6 that was used as a BCG booster vaccine. It could elevate antigen-specific IFN-γ secretion, but as for polyfunctional T cells they did not investigate. Jia et al. (12) reported an attenuate LM recombinant strain expressing a *Mtb* protein r30. It could boost BCG-immunized mice and guinea pigs to induce higher polyfunctional CD4^+^ T lymphocytes but not CD8^+^ T cells.

In this report, results showed that BCG-primed mice boosted with LMΔ-*msv* and LIΔ-*msv* possessed significant resistance to mycobacterial infection in the lung and spleen (Figures 6A,B). Such mice showed the lightest pulmonary granulomatous consolidate (Figures 6F–I) as well as the lowest level of SP-A and ADA in serum and lung (Figures 6C–E). Some TB vaccine candidates were constructed as boosters of BCG, and some of them demonstrated had better efficacy than BCG alone in mice, guinea pigs, non-human primates and cattle (6, 11, 12, 19, 45). Yin et al. (11) reported an attenuated LM-vectored TB vaccine that was used as a booster vaccine for BCG, which could confer higher protection against *Mtb* challenge than BCG, with decreasing the bacterial load in the lung but not the spleen. Jia et al. (12) reported that intradermal vaccination with attenuated LM-vectored TB vaccine could improve protective efficacy in BCG-immunized hosts. As the protective efficacy of TB vaccines in animal models may be influenced by many factors, such as the vaccination schedule, the challenge strain, and the species of animals, it is hard to compare the efficacy of different type of vaccines.

In conclusion, our research provided a novel heterologous vaccination regimen that combined both LM and LI based vaccines as a compensate for the limitations of BCG. As a booster strategy for BCG, it could induce better protection efficacy than BCG alone. The protective efficacy could be attributed to multistage antigen-specific CD4^+^ T cell responses and strong multifunctional CD8^+^ T cell responses. These findings suggested that LM and LI vectored vaccination strategy may be a promising vaccine candidate that needs to be tested in future clinical trials. In our study, we used the lethal dose of BCG to test the protection efficacy of vaccine candidates before testing in BSL three facilities using virulent *Mtb*. It is an ongoing study. In addition, further studies should be carried out to test the effects of this multistage antigen vaccine in preventing the reactivation of dormant *Mtb* or therapy the LTBI. And the important role of CD8^+^ T cells in the protection of reactivation of LTBI is also needed to be studied in LTBI animal model.

## Data Availability Statement

The original contributions presented in the study are included in the article/Supplementary Material, further inquiries can be directed to the corresponding authors.

## Ethics Statement

The animal study was reviewed and approved by Animal Care and Use Committee of Sichuan University.

## Author Contributions

CW and X-YF: conceptualization and methodology. TT: validation. S-JL: formal analysis and writing–original draft preparation. S-JL, S-CT, and Y-WZ: investigation. J-MZ: data curation. CW: writing–review and editing and funding acquisition. All authors contributed to the article and approved the submitted version.

## Conflict of Interest

The authors declare that the research was conducted in the absence of any commercial or financial relationships that could be construed as a potential conflict of interest.
