# Supplementary material for: Heterologous Boosting With Listeria-Based Recombinant Strains in BCG-Primed Mice Improved Protection Against Pulmonary Mycobacterial Infection
Source: Front Immunol. 2020 Sep 2;11:2036. doi: 10.3389/fimmu.2020.02036 (PMC7492678; doi:10.3389/fimmu.2020.02036)

**Supplementary material**

**Fig. S1** Schematic overview of antigen gene *msv*. T-cell epitopes of *Rv2460c*, *Rv2660c*, *Rv3875*, and *Rv3804c* were tandem-linked through linker (GCCGCCTAC) (as shown in black). The fusion gene was optimized to be expressed by *Listeria monocytogenes* 10403s. Then the *msv* sequence was ligated into a pUC57-simple vector, resulting in pUC57-*msv*.

**Fig. S2** Construction of targeting plasmids pCW702-*msv* and pCW154-*msv*. (A) The plasmid pCW203-*msv* was constructed by ligation of *msv* in *Hin*d Ⅲ and *Xho* Ⅰ restriction enzyme sites. (B) The *Bam*H Ⅰ-*Xho* Ⅰ fragment from pCW203-*msv* containing the HA epitopes (TPTAVPATA) and *msv* gene was ligated into pCW702-*msv* and pCW154-*msv* respectively, resulting in targeting plasmids pCW702-*msv* and pCW154-*msv.*

**Fig. S3** The standard curve of qPCR for BCG. Extracted genomic DNA of BCG from 10-fold serial dilution of BCG suspensions and CFU of the same BCG aliquots were quantified by solid cultured. Bacterial genomic DNA was extracted from each 1 mL of diluted suspension, and was eluted in a final volume of 50 μL. 2 μL of each 50 μL DNA sample was subsequently used for each PCR reaction. The standard curve was obtained by the Ct (cycle threshold) values and the Log (CFU) of BCG. The PCR limits of CFU detection in a 1 mL tissue homogenate therefore ranged from102.45 CFU to 108.45 CFU (R^2^=0.999).

**Fig. S4** Prokaryotic expression and immunogenicity analysis of fusion protein msv-His. (A) Western blotting analysis of purified msv-His protein. The *msv* gene was cloned into a prokaryotic expression vector pEASY-Blunt E1. The fusion protein was expressed under the induction of IPTG. Purified protein was identified by western blotting using the anti-His monoclonal antibody as primary antibody. (B) Specific anti-msv antibodies in msv protein immunized mice. C57BL/6J (7 mice/each group) were intraperitoneally immunized with protein msv/ Freund′s adjuvant or Freund′s adjuvant three times at week of 0, 1 and 2. Serum was collected every week, and antibody titers against msv were detected by ELISA. (C) Splenic lymphocyte proliferation in msv protein immunized mice. 4 weeks after the first immunization, spleens were collected and the splenic lymphocyte proliferation was assayed using MTT agent after stimulating with msv protein. Each point represents the mean ± SEM for a group of seven mice from one independent experiment. **P*＜0.05, ***P*＜0.01 and ****P*＜0.001.

**Fig. S5** PCR identification of the recombinant strains. Amplification of *msv* gene (**A**) and *ery* gene from LMΔ-*msv* **(1)**, LIΔ-*msv* **(2)**, negative control **(N),** and positive control **(P)**.

**Fig. S6** The survival curves of mice after intravenous injection with the *Listeria* strains. C57BL/6J mice (10 mice per each group) were injected with a gradient-increased dose of LMΔ-*msv* (A), LM wild type strain (B), LIΔ-*msv* (C) and LI wild type strain (D). Then the mice were monitored for the next 10 days.

**Fig. S7** The AST level in serum of mice infected with recombinant strains. Mice were infected with 10^6^ CFU of LM△-*msv* or 10^7^ CFU of LI△-*msv*. Serum were harvested 1, 2, 3, 5, 7, and 14 dpi, and detected by an automatic biochemical analyzer. Results were presented as mean ± SEM per group of nine mice. All the experiments were repeated three times.

**Fig. S8** Schedule for mice immunization and immune responses detection. (A) C57BL/6J mice (10 mice per each group) were intravenously immunized with LMΔ-*msv*, LIΔ-*msv*, LMΔ-*lacZ*, LIΔ-*lacZ* or normal saline respectively. Nine days later, five mice of each group were sacrificed for detecting cellular immune responses. Other mice were boosted with the same strain two weeks after the first immunization. Two weeks after the last immunization, serums were collected for detecting the total IgG titers of anti-msv proteins. (B) C57BL/6J mice were intravenously immunized with LMΔ-*msv* or LIΔ-*msv*, and 40 days later, they were boosted with the same strain or another strain. Nine days after the last immunization, the T cell responses were determined by flow cytometry.

**Fig. S9** Schedule for mice immunization and BCG challenge. Nine days after the last immunization, spleen T cell responses were determined. Four weeks after the last immunization, mice were intranasally infected with 1×10^7^ CFU of BCG. Three weeks post challenge, the mice were sacrificed and the numbers of BCG in the lung and spleen were determined by real-time RT-PCR. The histopathological changes of lung were determined by ELISA and lung tissues HE-stained.

**Fig. S10** Boosting with LMΔ-*msv* → LIΔ-*msv* increased T cell responses primed by BCG immunization. C57BL/6J (10 mice/group) primed by BCG were boosted with LMΔ-*msv* → LIΔ-*msv* or not. Splenocytes were collected 9 days after the last inoculation, stimulated for 5 h with PPD (10 μg/mL), and analyzed for cytokine production by ICS assay as described in Fig 3**.** (**A**) Characterization of poly-functional T cell responses in the spleen. The proportions of CD4^+^ or CD8^+^ T cells that produce one, two or three cytokines are shown in the bar graph. (**B**) The proportion of the three types of CD4^+^ and CD8^+^ T cells are shown in the pie chart. All the experiments were performed with biological triplicates. Each point represents the mean ± SEM for a group of seven mice from one independent experiment. **P*＜0.05, ***P*＜0.01 and ****P*＜0.001.

**Fig. S11** Full-length images of the Western blotting blots. (**A** and **B**) Western blotting of culture supernatant and cell lysate from LMΔ-*msv* (**A**) and LIΔ-msv (**B**) using the anti-HA monoclonal antibody as primary antibody. The protein in the supernatant of LMΔ-*msv* (lane 1), LMΔ-*lacZ* (lane 3), LIΔ-*msv* (lane 5) and LIΔ-*lacZ* (lane 7). The protein in the cell lysates of LMΔ-*msv* (lane 2), LMΔ-*lacZ* (lane 4), LIΔ-*msv* (lane 6) and LIΔ-*lacZ* (lane 8). (**C**) Western blot of cell lysates using an anti-HA monoclonal antibody to detect the fusion msv protein. RAW264.7 cells were uninfected or infected at a MOI of 100:1 with LMΔ-*msv* or LIΔ-*msv.* At 7 h post infection, the infected cells were lysed and subjected to Western blot. Protein of cells infected with LMΔ-*msv* (lane 9), LIΔ-msv (lane 10) and NS (lane 11).

**
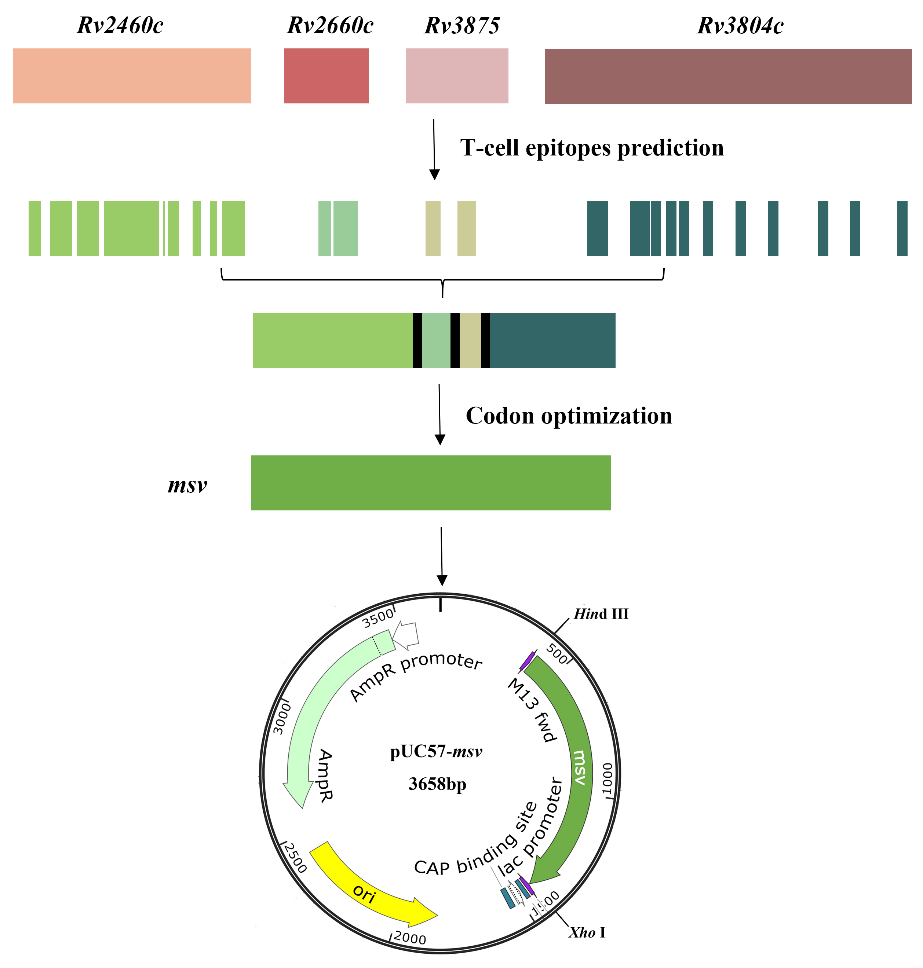
**


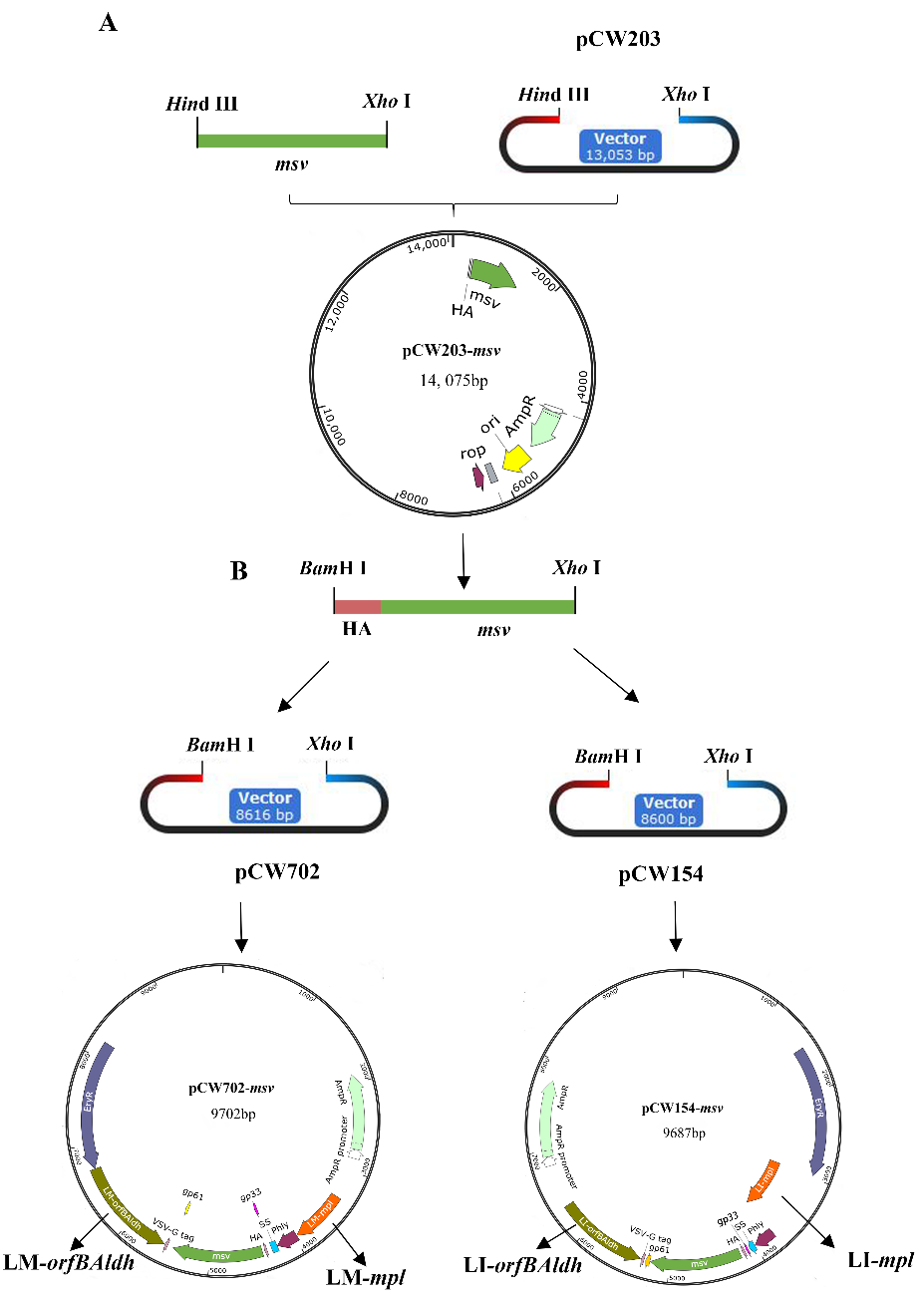


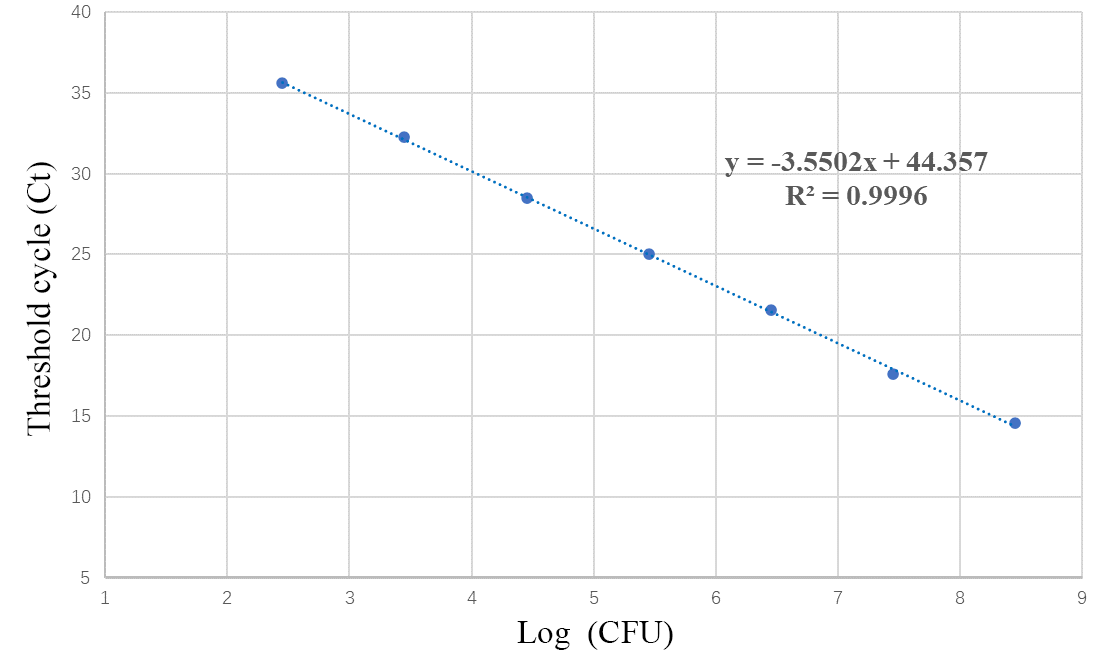


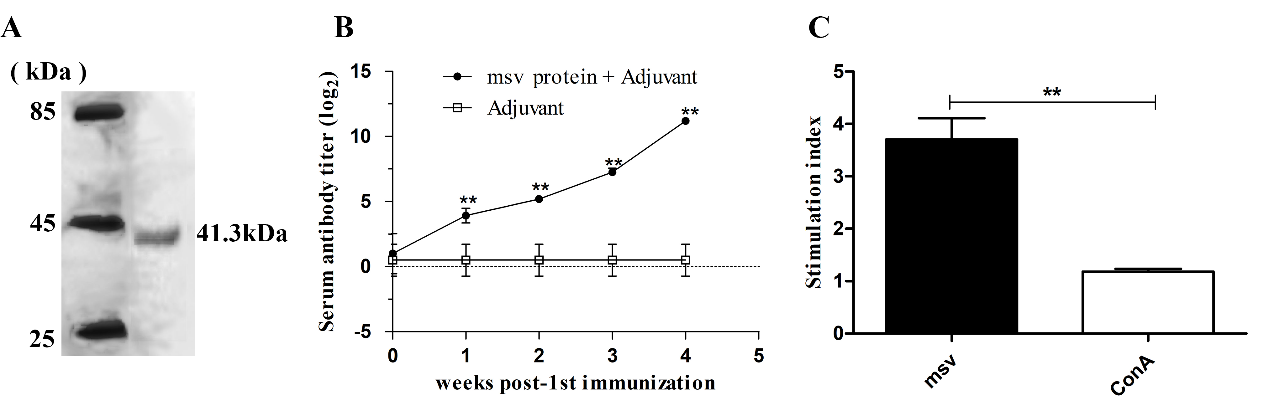


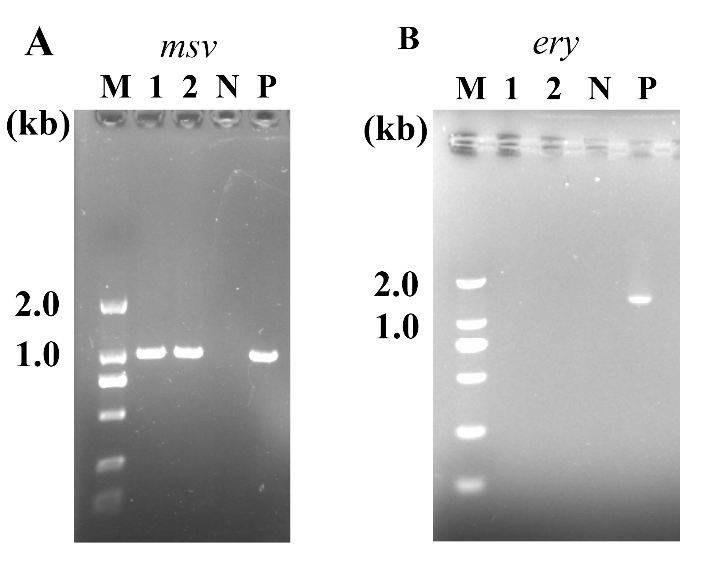


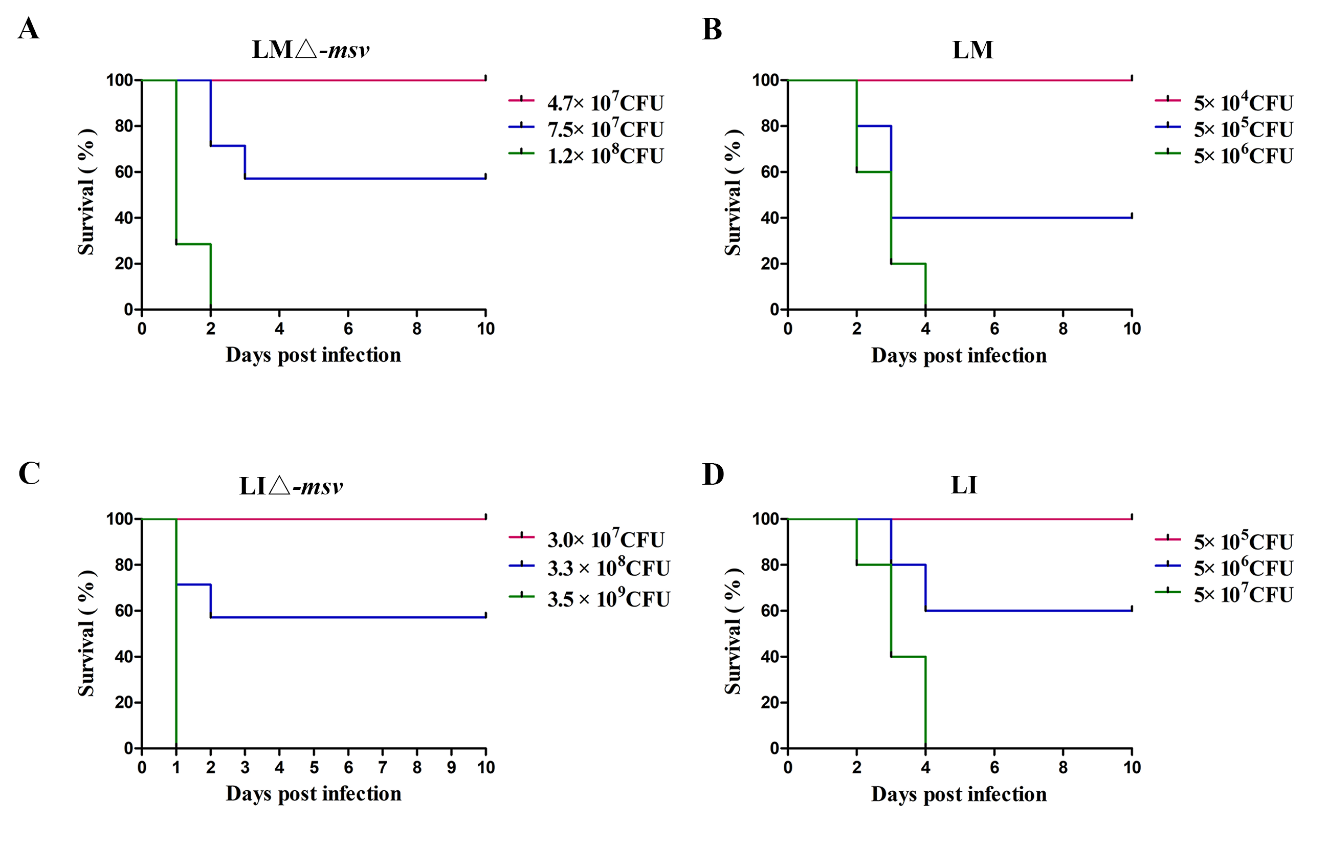


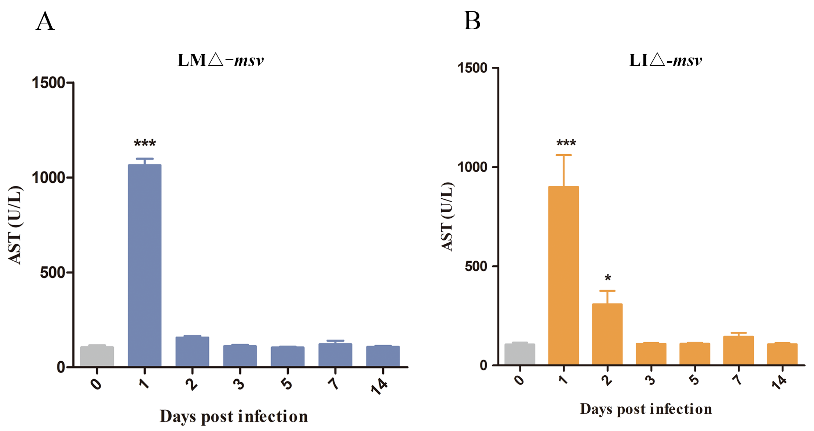


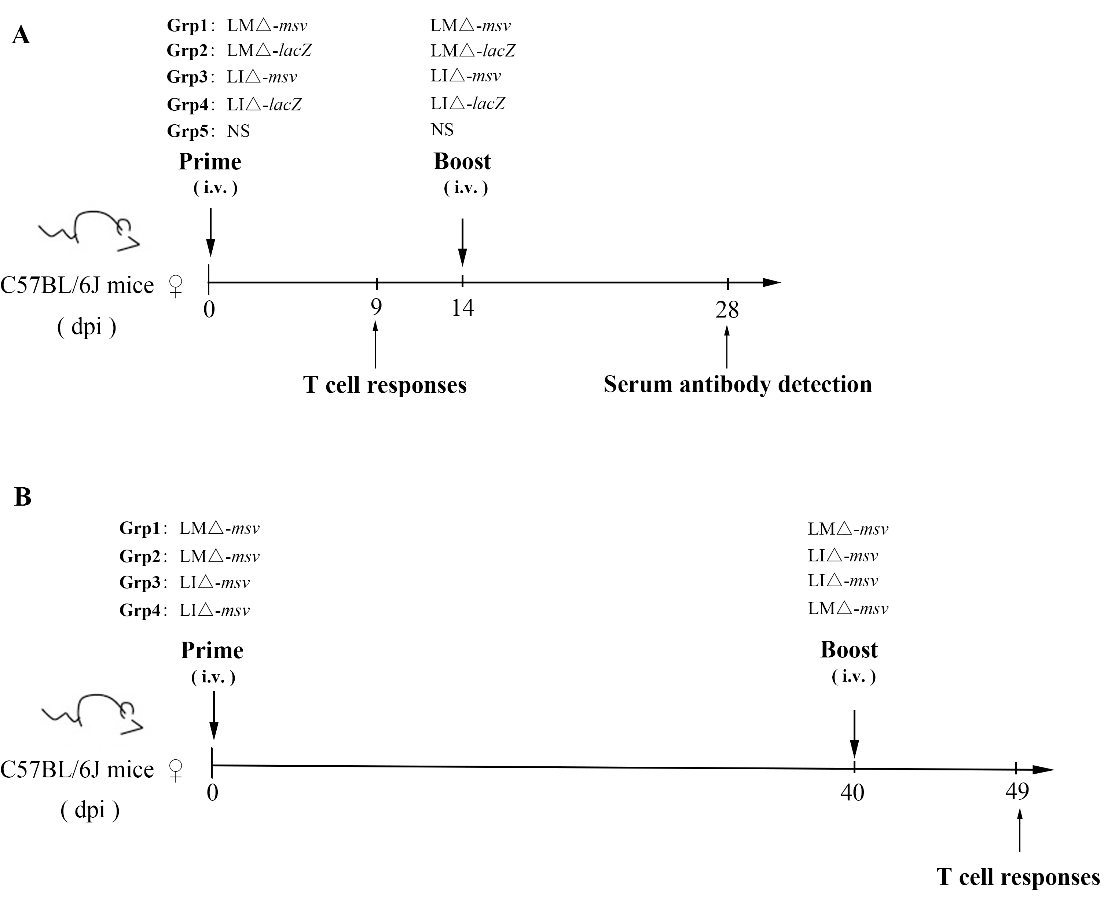


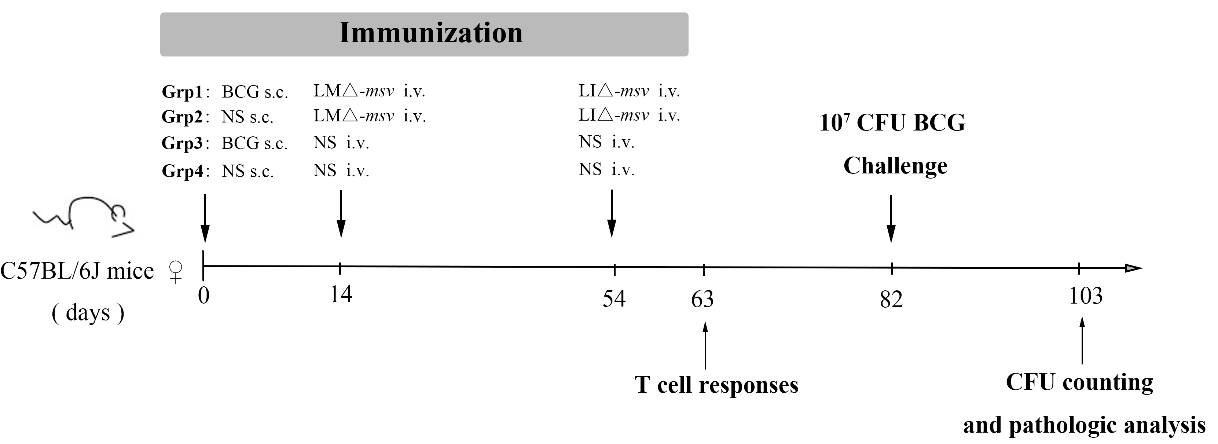


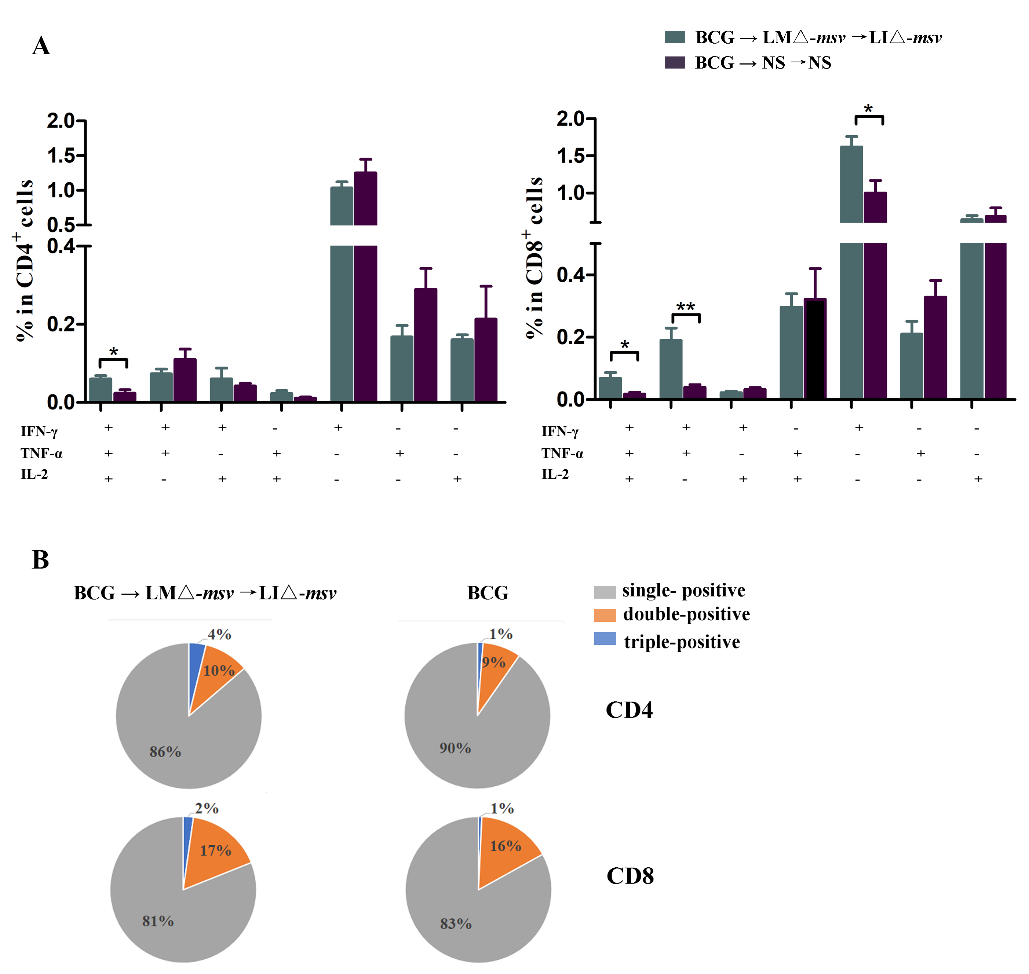


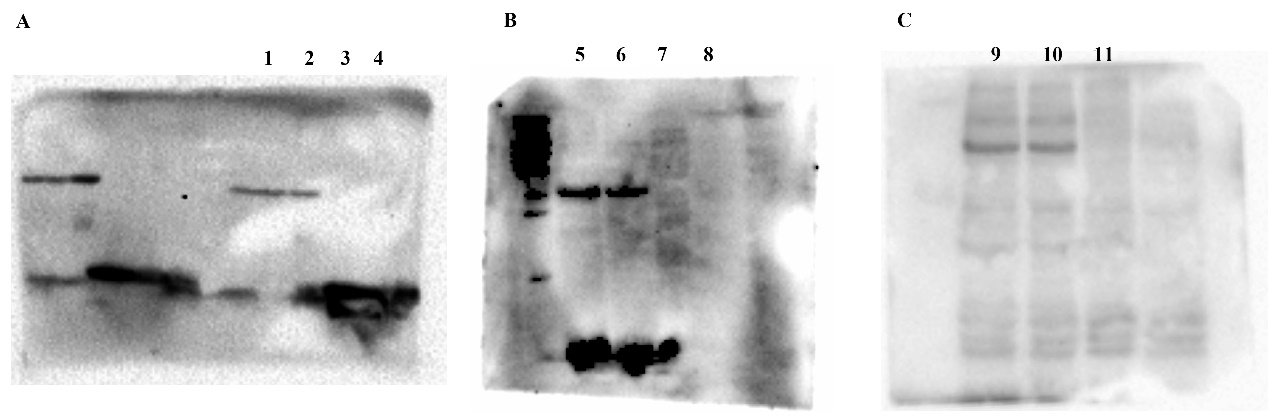

Supplement: Supplementary file 1 [file Data_Sheet_1.docx]
